# The Helminths Causing Surgical or Endoscopic Abdominal Intervention: A Review Article

**Published:** 2017

**Authors:** Erdal UYSAL, Mehmet DOKUR

**Affiliations:** 1. Dept. of General Surgery, School of Medicine, Sanko University, Gaziantep, Turkey; 2. Dept. of Emergency, Necip Fazil City Hospital, Kahramanmaras, Turkey

**Keywords:** Helminths, Surgery, *Ascaris*, Endoscopy, *Enterobius*, *Taenia*

## Abstract

**Background:**

Helminths sometimes require surgical or endoscopic intervention. Helminths may cause acute abdomen, mechanical intestinal obstruction, gastrointestinal hemorrhage, perforation, hepatitis, pancreatitis, and appendicitis. This study aimed to determine the surgical diseases that helminths cause and to gather, analyze the case reports, case series and original articles about this topic in literature.

**Methods:**

This study was designed as a retrospective observational study. In order to determine the studies published in literature, the search limits in Pub-Med database were set to 1 Jan 1957 and 31 Mar 2016 (59 yr), and the articles regarding Helminth-Surgery-Endoscopy were taken into examination. Among 521 articles scanned, 337 specific ones were involved in this study.

**Results:**

The most common surgical pathology was found to be in Ascaris lumbricoides group. *Enterobius vermicularis* was found to be the parasite that caused highest amount of acute appendicitis. Anisakiasis was observed to seem mainly because of abdominal pain and mechanical intestinal obstruction. *Strongyloides stercoraries* causes duodenal pathologies such as duodenal obstruction and duodenitis. *Taenia saginata* comes into prominence with appendicitis and gastrointestinal perforations. *Fasciola hepatica* exhibits biliary tract involvement and causes common bile duct obstruction. Hookworms were observed to arise along with gastrointestinal hemorrhage and anemia. *Trichuris trichiuria* draws attention with gastrointestinal hemorrhage, mechanical intestinal obstruction.

**Conclusion:**

Helminths may lead to life-threatening clinic conditions such as acute abdomen, gastrointestinal perforation, intestinal obstruction, and hemorrhages. There is a relationship between surgery and helminths. It is very important for surgeons to consider and remember helminths in differential diagnoses during their daily routines.

## Introduction

Helminths exist widely throughout the world. All of the systems within any organism may be affected from the helminths. From the simple infestations to the life-threatening wide involvements, they can be observed in wide spectrum (1, 2). While medical treatment is enough for some of the pathologies caused from helminths, some others may require surgical or endoscopic intervention. Surgical treatment is generally related with gastrointestinal system. In previous studies, *Ascaris lumbricoides*, *Anisakis* spp, *E. vermicularis, Strongyloides stercoralis, Taenia saginata, Fasciola hepatica, N. americanus, Ancylostoma duodenale, Trichuris trichiura* and rarely *W. bancrofti* have been observed to cause surgical or endoscopic intervention (3–5). These parasites exhibit their effects initially by residing at digestive system. Helminths lead to acute abdomen, mechanical intestinal obstruction, gastrointestinal hemorrhage, gastrointestinal perforation, obstructive jaundice, pancreatitis, and appendicitis (6, 7). Moreover, some of the helminths establish tumor-like lesions and mimic the tumors (8). In some of the studies, the helminths might be related to tumors (9, 10). In such clinic conditions, the patients require surgical or endoscopic interventions.

In literature review, majority of the studies on surgery-related helminths consist of case reports or case series from different centers worldwide. There is a limited number of studies on specific topics such as biliary tract migration or helminths, their relation with tumor, and appendicitis. There is not a sufficient number of studies, where the helminths causing surgical or endoscopic intervention are presented together.

In our study, it was aimed to determine the surgical diseases that helminths cause, and to gather and analyze the case reports, case series, and original articles regarding the surgical diseases caused from helminths.

## Methods

### Study Design and Literature search

Our study was designed as a retrospective observational study. In order to find the studies published in international literature, the search parameters for PubMed database were set to period between 1 Jan 1957 and 31 Mar 2016 (59 yr), and the publications related with Helminth-Surgery-Endoscopy were taken into consideration. PubMed database was selected, because it is widely used throughout the world, and consists of 5400 specifically selected medical journals in 39 languages. Of 521 articles scanned in total, 337 articles were involved in this study. As the research methodology, the articles were clustered to 3 main titles, namely original research articles, case series, and case reports. Of these 337 articles, 17 articles were classified into original article, 40 into case series, and 279 into case reports. The complete texts of all the articles were accessed, and the information obtained from PubMed was completed from these texts. The analysis of the data obtained was executed independently by two researchers.

### Inclusion and excluding criteria

The articles involving parasites caused surgical or endoscopic intervention or been related with surgical diseases were involved in this study. The articles not related to surgical and endoscopic intervention or surgical diseases, the surgical pathologies that *Echinococcus granulosus* has caused, and the experimental studies were not involved.

### Statistical Data Analysis

Surgical pathologies, which helminths caused, and their diagnoses and treatments were recorded. No statistical comparison was performed in this study. The pathologies caused from helminths leading to surgical or endoscopic intervention were classified in tables.

## Results

In our study, *A. lumbricoides, Anisakis, S. stercoralis, T. saginata, F. hepatica, E. vermicularis, N. americanus, Ancylostoma duodenale, T. trichiura* and rarely *W. bancrofti* were found to cause surgical or endoscopic intervention. *A. lumbricoides* was found to cause the most common surgical pathology.

### Ascaris lumbricoides

*A. lumbricoides* was found to migrate to biliary tract and then cause pathologies such as hepatitis and obstruction in biliary tract. A total of 84 case reports, 12 case series, and 6 original articles were related to the surgical pathologies that *A. lumbricoides* causes.

### Anisakis

*Anisakis* was observed to seem mainly because of adnominal pain and mechanical intestinal obstruction. A total of 39 case reports, 8 case series, and 3 original articles were related to the surgical pathologies that *Anisakis* causes.

### Strongyloides stercoralis

*S. stercoralis* was observed to lead to wide spectrum of surgical pathology. *S. stercoralis* causes the duodenal pathologies such as duodenal obstruction and duodenitis.

Moreover, it was determined to lead to hyper-infection syndrome in patients receiving immunosuppressive medication after solid organ transplantation and having immune deficiency. A total of 37 case reports, 3 case series, and 1 original article were related to the surgical pathologies that *S. stercoralis* causes.

### Enterobius vermicularis

*E. vermicularis* was found to be the helminth that caused the highest amount of acute appendicitis. A total of 23 case reports, 9 case series, and 6 original articles were related to the surgical pathologies that *E. vermicularis* causes.

Among them, 23 articles, 6 of which were original, were found to be related with acute appendicitis.

### Fasciola hepatica

*F. hepatica* involves in biliary tract and causes common bile duct obstruction. A total of 34 case reports and 4 case series were related to surgical pathologies that *F. hepatica* causes.

### Taenia saginata

*T. saginata* was seen to cause especially acute appendicitis and also gastrointestinal perforations and migration to biliary tract. A total of 34 case reports and 2 case series were related to the surgical pathologies that *T. saginata* causes.

Hookworms were observed to come into prominence with gastrointestinal hemorrhage and anemia.

### Trichuris trichiuria and Wuchereria bancrofti

Totally 12 case reports and 1 case series were found related to surgery pathologies that hook worms cause. *T. trichiuria* draws attention with gastrointestinal hemorrhage, mechanical intestinal obstruction, colon perforation, and inflammatory lesions in colon. Totally 6 case reports were found to be related to surgical pathologies that *T. trichiuria* cause. *W. bancrofti* was observed to come into prominence with the pathologies in liver and lymphoma and its comorbidity with gallbladder cancer.

*Schistosoma subtype* was found to cause acute appendicitis (Table 1–6). The lowest number of articles (only 1 article) was found regarding *A. ceylanicuma*.

**Table 1:** Surgical pathology caused by *Ascaris lumbricoides*

**Pathology**	**Notifications**		
	**Case report**	**Case series**	**Orginal Article**
Small bowel anastomotic breakdown	1	-	-
Pancreatitis	8	-	-
Acute abdomen	2	-	-
Appendicitis	1	1	-
Migration to the biliary tract	18	6	6
Biliary tract obstruction	6	-	-
Cholangitis	1	-	-
Gastrointestinal bleeding	2	-	-
Intestinal perforation	3	-	-
Mechanical intestinal obstruction	5	1	-
Small bowel volvulus	3	-	-
Postoperative complications			
• Nasogastric tube obstruction	2	-	-
• Inflammatory injury in bowel Wall	1	-	-
• Laryngeal spasm	2	-	-
• Obstruction of Kehr’s drain	1	-	-
• Intraperitoneal migration	1	-	-
• Chest tube obstruction	1	-	-
• Cause of postcholecystectomy symptoms	1	-	-
• Stump cholecystitis	1	-	-
• Obstruction of jejunostomy tube	1	-	-
Mimicking gallbladder cancer	1	-	-
Ureteral colic	1	-	-
Hematuria	1	-	-
Hepatic abscess	1	2	-
Epigastric pain	3	-	-
Gallbladder ascariasis	2	1	-
Biliary tract stone	3	1	-
Stomach infestation	4	-	-
Togetherness with tumor			
• Villous tumor of the ampulla of Vater	1	-	-
• Pancreatic tumor during EUS	1	-	-
• Pancreas inflammatory tumor	1	-	-
Neobladder perforation	1	-	-
Meckel’s diverticulitis	2	-	-
Metallic biliary stent obstruction	1	-	-
Total	84	12	6

EUS: Endoscopic ultrasonography

**Table 2:** Surgical pathologys caused by *Enterobius vermicularis*

**Pathology**	**Notifications**		
	**Case report**	**Case series**	**Orginal Article**
Appendicitis	8	9	6
Mimicking Crohn’s disease	1	-	-
Intestinal perforation	1	-	-
Intestinal obstruction	1	-	-
Subcutaneous abscess	1	-	-
Liver Granuloma	1	-	-
Rare Locations			
• The kidney	1	-	-
• The Fallopian tube	1	-	-
• Meckel’s diverticulum	1	-	-
Togetherness with a tumor			
• Colon Tumor	1	-	-
• Neuroendocrine tumours	1	-	-
Inflammatory polyps	1	-	-
Isolated Abdominal Pain	2	-	-
Bartholin gland abscess	1	-	-
Ileal and colonic ulceration	1	-	-
Total	23	9	6

**Table 3:** Surgical pathology caused by *Anisakiasis*

**Pathology**	**Notifications**		
	**Case report**	**Case series**	**Original Article**
Abdominal pain	2	-	-
Togetherness with a tumor			
• Colon carcinoma	2	-	1
• Gastric carcinoma	1	-	1
Gastric anisiakiasis	7	4	-
Gastric ulcer in a Billroth II patient	1	-	-
Reflux esophagitis	1	-	-
Mechanical intestinal obstruction	8	1	-
Small bowel strangulation	1	-	-
Gastrointestinal bleeding	1	-	-
Acute Abdomen	5	2	-
Mesenteric ischemia	1	-	-
Assitis	2	-	-
Mimicting tumor			
• Gynecological cancer on PET-CT	1	-	-
• Gastric anisakiasis presenting as a submucosal tumour	1	-	-
• Presenting as an obstructive duodenal tumor	1	-	-
• Early gastric cancer superimposed	1	-	-
• Gastric stromal tumors	1	-	-
Appendicitis	1	-	-
Ileitis, colitis	-	1	-
Rectal polyp	1	-	-
Intestinal mucosal changes	-	-	1
Total	39	8	3

**Table 4:** Surgical pathology caused by Hook worms, *Fasciola hepatica* and *Taenia saginata*

**Pathology**	**Notifications**		
	**Case report**	**Case series**	**Orginal Article**
**Hook worms**			
Chronic diarrhoea	1	-	-
Severe Anemia	1	-	-
Gastric localization	2	-	-
Gastrointestinal Bleeding	6	1	-
Histopathological changes	1	-	-
Colitis	1	-	-
***Fasciola hepatica***			
Ectopic (Mesocolon)	1	-	-
Cholangitis	1	-	-
Liver mass	4	-	-
Obstructive jaundice (common bile duct obstruction)	9	2	-
Biliary fascioliasis	13	2	-
Hemobilia	1	-	-
Extrahepatic cholestasis	2	-	-
Mimicking a peritoneal carcinomatosis	1	-	-
Pancreatitis	1	-	-
Gallbladder fascioliasis	1	-	-
***Taenia saginata***			
Gastrointestinal perforation	5	-	-
Gallbladder Perforation	1	-	-
Intestinal obstruction	2	-	-
Colonic anastomosis leakage	1	-	-
Duodenal stump leakage	2	-	-
Esophageal leak	1	-	-
Extraluminal manifestation	2	-	-
Acute appendicitis	9	2	-
Acute cholangitis	1	-	-
Acute acalculous cholecystitis	1	-	-
Acute gangrenous cholecystitis	1	-	-
Acute pancreatitis	1	-	-
Meckel’s diverticulitis	1	-	-
Acute intestinal bleeding	1	-	-
Abnormal vaginal bleeding	1	-	-
Acute abdomen	1	-	-
Migration of biliary tract	3	-	-
Total	80	7	-

**Table 5:** Surgical pathology caused by *Strongyloides stercoralis*

**Pathology**	**Notifications**		
	**Case report**	**Case series**	**Orginal Article**
Mechanic intestinal obstruction	1	-	-
Subileus	2	-	-
Gastrointestinal bleeding	4	-	-
Duodenitis	2	-	-
Colitis	1	-	-
Duodenal mucosal nodularity	1	-	-
Gastric infection	3	1	-
Mimicking a malignant tumor	2	-	-
Obstructive duodenal stenosis	4	-	-
Pancolitis	-	-	1
Ascites	1	-	-
Transplantation patients			
• Intestinal	1	-	-
• The pancreas	1	-	-
• The kidney	2	1	-
Togetherness with a tumor			
• Lymphoma.	3	-	-
• Pancreatic cystadenocarcinoma	1	-	-
Mimicking ulcerative colitis	1	1	-
Fatal cutaneous Strongyloidiasis	1	-	-
Bilateral parotid abscesses	1	-	-
Biliary obstruction	1	-	-
Gastric perforation	1	-	-
Acute pancreatitis	3	-	-
Total	37	3	1

**Table 6:** Surgical pathology caused by other other helminths

**Pathology**	**Notifications**		
	**Case report**	**Case series**	**Orginal Article**
***Trichuris trichiuria***			
• Inflammatory responses in the colon	1	-	-
• Sessile polyp of the colon	1	-	
• Massive infestation of the intestinal mucosa	1	-	-
• Caecal ulcerative inflammation	1	-	-
• Colon obstruction and perforation	1	-	-
• Gastrointestinal bleeding	1	-	-
***Angiostrongylus costaricensis***			
• Pseudoneoplastic lesions (colon)	1	-	-
• Mimicking acute appendicitis	1	-	-
***Wuchereria bancrofti***			
• Cystic liver lesions	1	-	-
• Accompanied by gallbladder cancer	1	-	-
• Accompanied by cavernous hemangioma	1	-	-
• Association with adrenal lymphoma	1	-	-
***Schistosoma haematobium***			
• Rectal inflammatory polyp	1	-	-
• Cancer of urinary bladder	-	-	1
***Ancylostoma ceylanicum***			
• Iron-deficiency anemia	1	-	-
***Schistosoma japonicum***			
• Eosinophilic appendicitis	2	-	-
***Other Schistosomial appendicitis***	-	1	-
Total	16	1	1

## Discussion

Helminths are widely seen around the world. All of the systems in organism can be affected from helminths. However, they mostly influence the gastrointestinal system. Some of the clinic tables that helminths cause surgery or endoscopic intervention. For this reason, it is very important in daily surgery practice to remember the parasites that may cause life-threatening clinic conditions.

*A. lumbricoides* was found to be the parasite causing highest number of surgical intervention. Furthermore, it is also seen to be the parasite having widest spectrum of pathology. *A. lumbricoides* is the second most widely seen intestinal parasite worldwide (11). Although Ascaris lumricoides can affect the entire intestinal system, it mainly migrates to biliary tract and causes bilious pathologies (3, 12). The main pathologies that *Ascaris lumricoides* causes are cholangitis, obstructive jaundice, pancreatitis, and bile calculus. *A. lumbricoides* may lead to liver apses in endemic regions (13, 14). It is superior to many of parasites in terms of atypical location. By locating on the chest tube, nasogastric catheter, metallic biliary stent, jejunostomy tube, and operation drains, it leads to obstructions (15, 16). It may rarely locate in gallbladder and stomach (17, 18). *A. lumbricoides* may rarely cause pathologies related with urinary system such as hematuria and ureter colic (19). Moreover, acute abdomen cases that *A. lumbricoides* caused have been reported (20, 21). Since it causes epigastric and recurrent abdominal pain, differential diagnosis should be considered. *A. lumbricoides* may also lead to acute appendicitis and Meckel diverticulitis (22). Mechanical intestinal obstruction is another important clinic condition that *Ascaris* leads. Emergency surgeries may be required because of the mechanical intestinal obstructions that *A. lumbricoides* causes (23). *Ascaris* should be kept in mind as a reason for upper digestive hemorrhage (24). *A. lumbricoides* may lead to intestinal gangrene and perforation (25). Besides the intestinal system, it may also cause lethal respiratory obstruction. In our study, totally 84 case reports, 12 case series, and 6 original articles were found to be related to the surgical pathologies that *A. lumbricoides* causes. Overall, 50 of these articles, 6 of which are original, were about the biliary tract and pancreas pathologies.

In our study, *E. vermicularis* was found to be the parasite that causes highest number of acute appendicitis (7, 26). *E. vermicularis* may, in small intestines, lead to infections like Crohn’s disease. Moreover, they also cause intestinal obstruction (27). In case of reports, they led to granuloma in liver and abscess in subcutaneous tissue (28). *E. vermicularis* may be rarely seen in atypical localizations such as kidneys and fallopian tubes (29). *E. vermicularis* has also been found in Meckel’s diverticulum (30). Totally 23 case reports, 9 case series, and 6 original articles were related to the surgical pathologies that *E. vermicularis* causes. Of these articles, 23 articles, 6 of which are original, were found to be related with acute appendicitis.

*Anisakis* infects via raw sea products and leads to symptoms and pathologies induced by immunoglobulin E. Clinical manifestations may vary from urticaria to anaphylaxis (31). Moreover, it may be seen as intestinal granulomas and infections like Crohn’s disease (32). *Anisakis* also leads to mechanical intestinal obstructions (33, 34). In some cases, without leading to complete obstruction in small intestines, it may cause stenosis. In patients having severe intestinal stenosis, which does not respond to conservative treatments, the early surgical intervention should be considered (35). A rare reason for mechanical intestinal obstruction may be the bands formed because of granulomatous reaction caused from *Anisakis* extra-gastrointestinal located in mesos of small intestines (36). Moreover, *Anisakis* is also a reason for gastrointestinal system hemorrhages (37). *Anisakis* can establish lesions mimicking the tumors (38). *Anisakis* may lead to acute abdomen (39). *Anisakis* larvae located in gut may cause acute gastric *Anisakis*. Acute gastric *Anisakis* is characterized with severe epigastric pain. Acute gastric *Anisakis* can be endoscopically diagnosed and treated successfully (40). In treatment of diseases that *Anisakis* leads, laparoscopy can also be successfully utilized (41). *Anisakis* has been shown to cause parasitic abscesses in digestive tract (42). Although it generally leads to infections in Far East, its incidence in Europe also increases (43). For this reason, *Anisakis* should be known by all the surgeons worldwide. In a study, of 18 patients undergone surgery due to pathologies caused from *Anisakis*, 14 patients have been found to have ileitis or colitis and 1 patient has been found to have mechanical intestinal obstruction and another one has been determined to have acute appendicitis (44). In our study, totally 39 case reports, 8 case series, and 3 original articles were related with the surgical pathologies that *Anisakis* causes. Of three articles evaluated in our study, two articles were related with its association with tumor and other one was related with the mucosal changes those *Anisakis* causes (45, 46).

*S. stercoralis* affects 100 million of people worldwide. It is one of the most important reasons for abdominal pain and diarrhea. It generally causes chronic and limited disease, while, in immunocompromised patients, hyperinfection syndrome may occur (47). *S. stercoralis* was seen to be associated especially with obstructive duodenal stenosis, duodenitis, and duodenal mucosal nodularity (48). Although it causes wide spectrum of pathology, the rare cases were reported to be intestinal perforation, gastrointestinal hemorrhage, mechanical intestinal obstruction, biliary obstruction, and acute pancreatitis (49–51). *S. stercoralis*, especially after the transplantation, may lead to hyperinfection syndrome (52). Hyperinfection syndrome sometimes leads to infections that may be lethal (53). *S. stercoralis* may seem through tumor-like masses in gastrointestinal system (54). It may rarely cause pathologies, which require surgical intervention, outside of the gastrointestinal system, such as parotid abscesses. Totally 37 case reports, 3 case series, and an original article were related to surgical pathologies that *S. stercoralis* is responsible. The original article evaluated in our study was about the panchondritis that *S. stercoralis* is responsible for (55).

*T. saginata* causes especially acute appendicitis and gastrointestinal perforations and also acute cholecystitis, pancreatitis, and gastrointestinal hemorrhage (56, 57). They have also been reported to cause anastomosis leakage in postoperative period. They can create cholangitis by migrating to biliary tract (58). Totally 34 case reports and 2 case series were related to the surgical pathologies that *T. saginata* caused. Case series were about the acute appendicitis that *T. saginata* led.

*F. hepatica* leads to pathologies in biliary tract by locating to biliary tree. It mostly causes common bile duct obstruction and lead to obstructive jaundice (59). Locations at mesocolon and peritoneal ectopic may be rarely seen (60). It may lead to liver mass (61). In our study, totally 34 case reports and 4 case series were determined to be about the surgical pathologies that *F. hepatica* caused. Of these articles, 26 case reports and 4 case series were about the biliary involvement of *F. hepatica*.

Hookworms may cause clinic conditions such as gastrointestinal bleeding, and severe anemia (62). Some cases requiring explorative laparotomy due to gastrointestinal hemorrhage have also been reported (63). *A. duodenale* also pathologically leads to intestinal mucosa alterations (64). Hook-worms may cause colitis. In our study, totally 12 case reports and a case series were about the surgical pathologies that hookworms caused.

*T. trichiuria* causes ulcerative lesions, inflammatory reactions, and pathologies characterized with sessile polyp in colon. *T. trichiuria* rarely requires surgical intervention. It has been reported to cause obstruction and perforation in colon (65). *T. trichiuria* is a rare cause for hemorrhages in gastrointestinal system (66). In our study, totally 6 case reports were found to be related to surgical pathologies that *T. trichiuria* caused.

*Schistosoma* subtype may especially lead to eosinophilic appendicitis (67). In our study, one case series and three case reports about the appendicitis caused by *Schistosoma* sub-type were reviewed. Moreover, *Schistosoma haematobium* has been found to be associated with rectal inflammatory polyp.

*W. bancrofti* has been correlated with benign lesions in liver such as cystic liver lesions, and cavernous hemangioma (68). Moreover, in a case report, *W. bancrofti* has associated with the gallbladder cancer (69). In another case report, it has been correlated with adrenal lymphoma. In our study, totally four of the reviewed case reports were about the surgical pathologies of *W. bancrofti*.

*Angiostrongylus costaricensis* can be seen as appendicitis-like clinic table or pseudoneoplastic lesions in colon (70). Totally two case reports were about the surgical pathologies related with *A. costaricensis*. In our study, *A. lumbricoides, E. vermicularis, W. bancrofti, Anisakiasis, S. stercoralis* and *Schistosoma haematobium* might associate with the malign tumors. *Ascaris* has been seen with pancreatic tumor (71). *S. stercoralis* has been correlated with lymphoma and pancreatic cystadenocarcinoma (72). In addition, *E. vermicularis* has been reported with a colon tumor in a case report and with neuroendocrine tumor in another case report (73, 74).

*Anisakis*, however, is the parasite that has been mostly charged with in terms of tumor correlation (75). In our study, three case reports and two original articles, where *Anisakis* has been correlated with colon and gastric cancer, were reviewed. In case of reports, *W. bancrofti* has been reported to associate with gallbladder cancer and adrenal lymphoma (69, 76). *Schistosoma haematobium* has been correlated with cancer of urinary bladder (77). Even though the helminths have been seemed to associate with malign tumors, it could not be completely elucidated if they were an etiological factor causing malign tumors (78). For this reason, in order to reveal the relations of helminths with cancer, further clinic and experimental studies are needed.

In diagnosis and treatment of pathologies that helminths caused, the colonoscopy, esophagogastroduodenoscopy and endoscopic retrograde cholangiopancreography (ERCP) are utilized. Especially the ERCP is used widely in biliary tract pathologies that *A. lumbricoides* causes. Under favor of ERCP, the parasites can be removed from the biliary tract (79). Moreover, the differential diagnosis of pancreas and biliary tract pathologies can be executed. It is also successfully used with laparoscopic approach in treatment of biliary ascariasis (80). The diagnosis of *T. trichiura*, *A. lumbricoides*, *E. vermicularis*, and *Anisakis* can be made via colonoscopy (81). Moreover, the diagnoses of hookworm infections such as *A. duodenale*, *N. americanus* and *A. ceylanicum* and the diagnoses of *F. hepatica* and *T. saginata* can be made endoscopy and they can be endoscopically removed (82–84). Furthermore, the diagnosis and treatment of gastrointestinal hemorrhages that helminths caused can be made endoscopically.

Knowing and remembering the surgical pathologies, which helminths cause or are related with, worldwide would decrease the mortality and morbidity rates. In addition to the regions, where the helminths are endemic, they may lead to pathologies requiring surgical intervention. The helminths should be known to have relation with surgery (85).

## Conclusion

The largest portion of the articles reviewed was about *Ascaris lumbricoides. A. lumbricoides* causes widest spectrum of surgical pathologies. *E. vermicularis* is seen to be the helminth causing acute appendicitis most frequently. *S. stercoralis* causes common infection after solid organ transplantations. *S. hematobium* has been closely correlated with gallbladder cancer. Helminths may lead to life-threatening clinic conditions such as acute abdomen, perforation, obstruction, and gastrointestinal hemorrhages. There is a relationship between surgery and helminths. It is very important for surgeons to consider and remember helminths in differential diagnoses during their daily routines.
